# Towards a standardized framework for AI-assisted, image-based monitoring of nocturnal insects

**DOI:** 10.1098/rstb.2023.0108

**Published:** 2024-06-24

**Authors:** D. B. Roy, J. Alison, T. A. August, M. Bélisle, K. Bjerge, J. J. Bowden, M. J. Bunsen, F. Cunha, Q. Geissmann, K. Goldmann, A. Gomez-Segura, A. Jain, C. Huijbers, M. Larrivée, J. L. Lawson, H. M. Mann, M. J. Mazerolle, K. P. McFarland, L. Pasi, S. Peters, N. Pinoy, D. Rolnick, G. L. Skinner, O. T. Strickson, A. Svenning, S. Teagle, T. T. Høye

**Affiliations:** ^1^ UK Centre for Ecology & Hydrology, Maclean Building, Benson Lane, Wallingford OX10 8BB, UK; ^2^ Centre for Ecology and Conservation, University of Exeter, Penryn TR10 9EZ, UK; ^3^ Department of Ecoscience and Arctic Research Centre, Aarhus University, C.F Møllers Alle 3, Aarhus, Denmark; ^4^ Department of Electrical and Computer Engineering, Aarhus University, C.F Møllers Alle 3, Aarhus, Denmark; ^5^ Center For Quantitative Genetics and Genomics, Aarhus University, C.F Møllers Alle 3, Aarhus, Denmark; ^6^ Centre d'étude de la forêt (CEF) et Département de biologie, Université de Sherbrooke, 2500 Boulevard de l'Université, Sherbrooke, Québec, Canada J1K 2R1; ^7^ Natural Resources Canada, Canadian Forest Service – Atlantic Forestry Centre, 26 University Drive, PO Box 960, Corner Brook, Newfoundland, Canada A2H 6J3; ^8^ Mila – Québec AI Institute, Montréal, Québec, Canada H3A 0E9; ^9^ Federal University of Amazonas, Manaus, 69080–900, Brazil; ^10^ The Alan Turing Institute, 96 Euston Road, London NW1 2DB, UK; ^11^ Naturalis Biodiversity Centre, Darwinweg 2, 2333 CR Leiden, The Netherlands; ^12^ Insectarium de Montreal, 4581 Sherbrooke Rue E, Montreal, Québec, Canada H1X 2B2; ^13^ Centre d'étude de la forêt, Département des sciences du bois et de la forêt, Faculté de foresterie, de géographie et de géomatique, Université Laval, Québec, Canada G1V 0A6; ^14^ Vermont Centre for Ecostudies, 20 Palmer Court, White River Junction, VT 05001, USA; ^15^ Ecole Polytechnique, Federale de Lausanne, Station 21, 1015 Lausanne, Switzerland; ^16^ Faunabit, Strijkviertel 26 achter, 3454 Pm De Meern, The Netherlands; ^17^ School of Computer Science, McGill University, Montreal, Canada H3A 0E99

**Keywords:** biodiversity monitoring, machine learning, moths, camera trap

## Abstract

Automated sensors have potential to standardize and expand the monitoring of insects across the globe. As one of the most scalable and fastest developing sensor technologies, we describe a framework for automated, image-based monitoring of nocturnal insects—from sensor development and field deployment to workflows for data processing and publishing. Sensors comprise a light to attract insects, a camera for collecting images and a computer for scheduling, data storage and processing. Metadata is important to describe sampling schedules that balance the capture of relevant ecological information against power and data storage limitations. Large data volumes of images from automated systems necessitate scalable and effective data processing. We describe computer vision approaches for the detection, tracking and classification of insects, including models built from existing aggregations of labelled insect images. Data from automated camera systems necessitate approaches that account for inherent biases. We advocate models that explicitly correct for bias in species occurrence or abundance estimates resulting from the imperfect detection of species or individuals present during sampling occasions. We propose ten priorities towards a step-change in automated monitoring of nocturnal insects, a vital task in the face of rapid biodiversity loss from global threats.

This article is part of the theme issue ‘Towards a toolkit for global insect biodiversity monitoring’.

## 1. Introduction

There is increasing attention on the combined environmental crises of climate warming and biodiversity declines, impacting nature's contributions to people's livelihoods across the planet [1]. In response, the Kunming-Montreal Global Biodiversity Framework (GBF) adopted during the fifteenth meeting of the Conference of the Parties (COP15) sets out an ambitious vision for ‘…*a world of living in harmony with nature where by 2050, biodiversity is valued, conserved, restored and wisely used, maintaining ecosystem services, sustaining a healthy planet and delivering benefits essential for all people*’ (https://www.cbd.int/gbf/vision). For nations to measure progress towards these targets we urgently need more effective ways of monitoring biodiversity [2]. Without more standardized and extensive data, we cannot fully understand and quantify the impacts of environmental change [3].

Meeting the GBF is particularly challenging for insects owing to their high diversity, estimated to make up 80% of all animal life [4], lack of baseline knowledge (e.g. an estimated 80% of species are yet to be described), scarcity of experts to identify species [5] and other technical challenges in monitoring their status [6]. Growing evidence of wholesale declines in insect diversity and abundance has drawn attention from researchers, policymakers and the wider public towards the importance of insects [7]. Similarly, climate change and increased globalization have led to an increased risk for the introduction of non-native species [8] and for native species to move beyond their existing ranges and niches [9]. Concern is heightened owing to the critical role that insects play in all terrestrial ecosystems as pollinators of crops and wild plants, vectors of disease and predators of crop pests, and for cycling of nutrients and for their cultural value.

Despite the clear need for a better understanding of trends in insect populations, there is a severe paucity of monitoring datasets and capacity for detection. To date, the evidence-base for insect declines remains biased to a few regions and a limited set of insect groups that are most tractable to monitor. Most assessments are restricted to a few well-studied groups of insects (e.g. primarily butterflies, and more recently other pollinators such as bees and flies), predominantly from Europe and North America [10]. More robust data on insects from diverse ecological guilds across the world are essential to address the wider challenges in entomology [11] and prioritize remedial action [12]. The need for more robust insect monitoring is particularly acute for biodiversity hotspots such as the tropics [13].

Recent development in technology offers new potential for biodiversity monitoring [14] to meet the growing need for long-term, high-resolution, spatially extensive and standardized biodiversity information [15]. For insects, the monitoring innovations showing the most potential are image analysis, acoustic monitoring, radar, and molecular methods [16]. No single approach can monitor all insects; a combination of technologies alongside traditional approaches will be required to enhance the spatial, temporal and taxonomic breadth of monitoring across the world.

Computer vision is one of the most scalable and fastest developing technologies for ecological applications, owing to the ready availability of sensors (e.g. digital cameras) combined with the potential to adapt machine learning methods initially developed for other fields. Compared with other technologies that are expensive or do not provide standardized abundance metrics, image-based monitoring of insects arguably has the greatest potential to track abundance at national levels as a contribution to global indicators [17]. Most monitoring of invertebrates continues to use established sampling approaches (e.g. point counts, transects and traps) although camera-based applications for insects are increasing rapidly [18]; almost 60% of camera-based studies for insects (J. Lawson, C. Grant, R. Ewers, J. Rosindell, W. Pearse 2024, in preparation) were published in the last 3 years.

Realizing the full potential of computer vision for monitoring insects requires systems that can be deployed in a range of field conditions. One approach to avoid the challenges of detecting and classifying insects on complex backgrounds (e.g. on vegetation or flowers) is the use of an attractant for insects (e.g. pheromone or light). Light-trapping camera systems have proven especially effective for detection of positively phototactic insects [19], particularly moths (a paraphyletic group comprising all Lepidoptera that are not butterflies), most of which are crepuscular or nocturnal. The ecological and taxonomic diversity of moths and their importance for ecosystem function make them an effective indicator group for the status of insects. For example, it is estimated that one in ten described species on Earth is a moth (160 000 moths out of 1.5 to 1.6 million described species), and there is increasing recognition of the importance of moths as pollinators that complement day-active species [20], the herbivory that they perform, and the sustenance that they provide for many terrestrial arthropods, birds and bats. A further advantage of an illuminated screen for detecting moths is simplification of computer vision applications, where featureless backgrounds and consistent lighting can improve algorithmic performance and reduce the complexity of localizing individuals [21]. Although at a relatively early stage of development, camera systems for nocturnal insects have proven feasible to implement and are likely to generate important data for assessing insect populations. For example, the DIOPSIS insect cameras have been used in The Netherlands since 2019, with over 150 cameras now deployed across the country [16]. To fully realize the wider potential of camera systems for nocturnal insects, there is a need for an operational and standardized framework.

**Figure 1.**
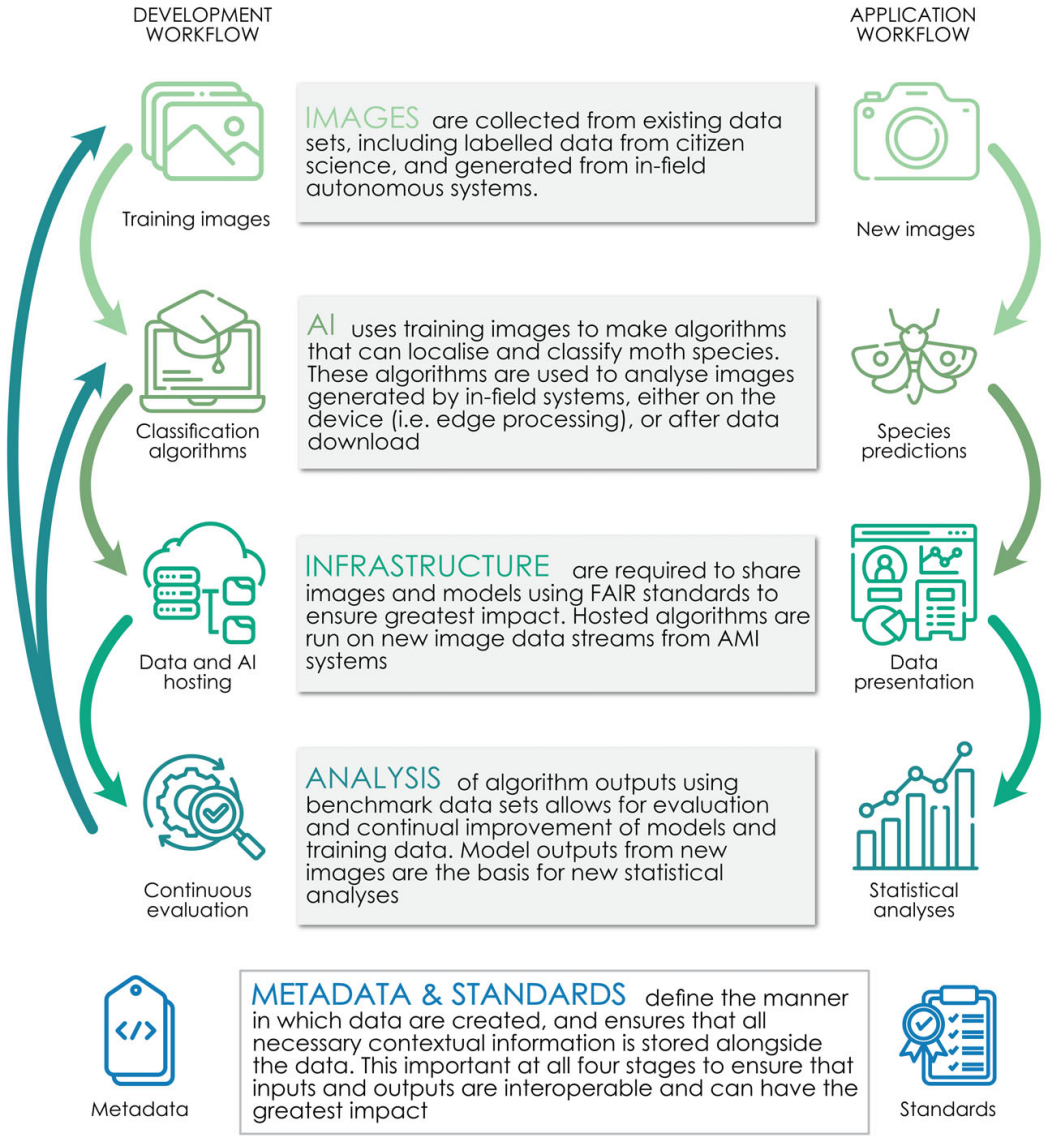
Schematic representation of the workflow for automated analysis of images from biodiversity sensors. AI, artificial intelligence.

In this paper, we describe the elements of a framework from sensor development and deployment to machine learning workflows for data processing, analysis and publishing (figure 1). Crucially, we identify priorities for future research and development to realize the huge potential of automated, artificial intelligence (AI)-assisted camera systems for monitoring insects. Developing automated sensors, alongside traditional approaches, is likely to make a major contribution to measuring progress towards national and international biodiversity targets, understanding the impacts of environmental change and identifying solutions to address the growing biodiversity crisis.

## 2. Deploying an automated camera system for monitoring nocturnal insects

**Figure 2.**
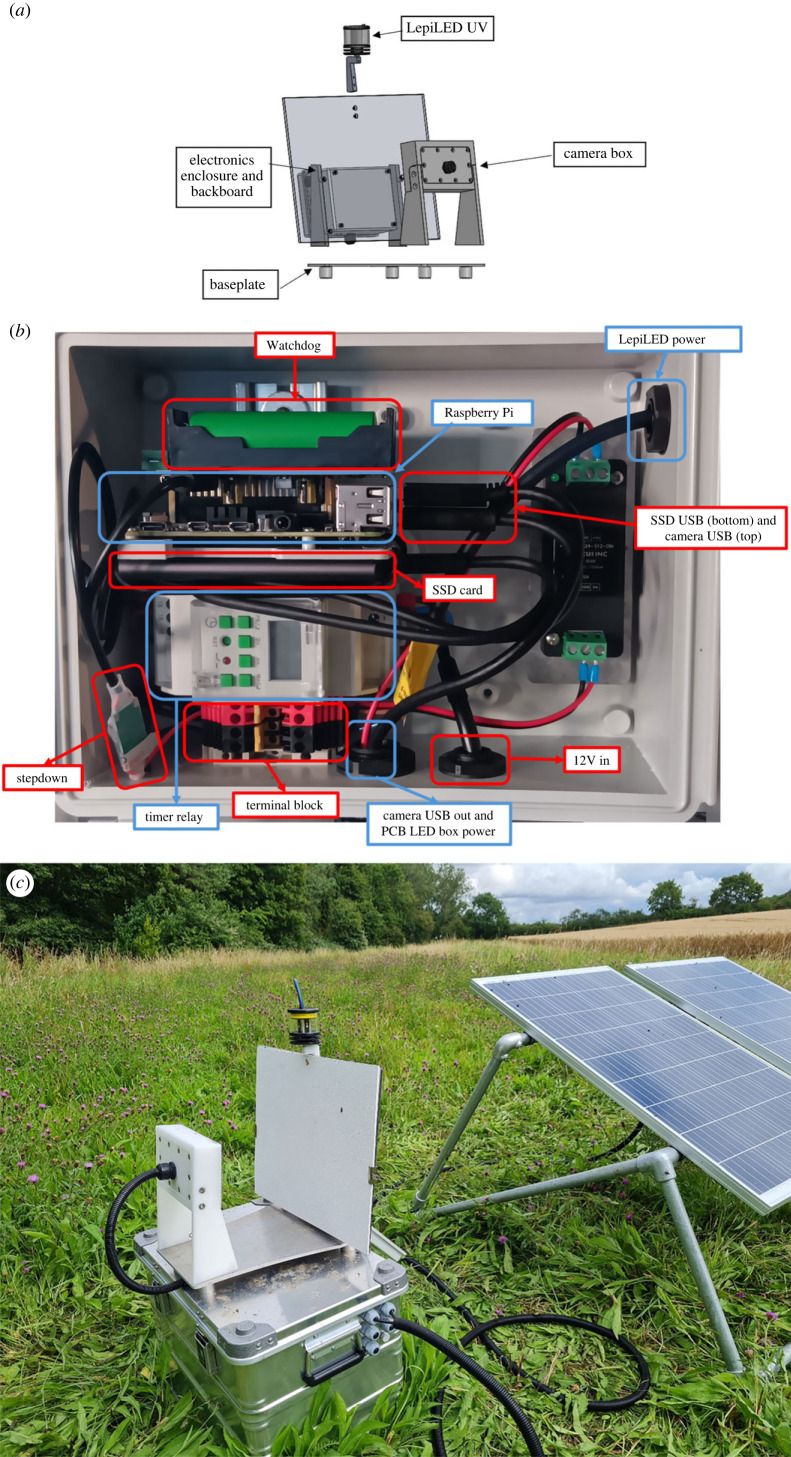
Automated Monitoring of Insects (AMI) system, illustrating the standard elements of an image-based sensor for nocturnal insects. (*a*) The portable light trap mounted on a baseplate and with a UV light to attract live moths during the night, a camera box with a light to illuminate a white board, and an enclosure containing a computer system and electronics. (*b*) Annotated photograph of computer and electrical components within a housing enclosure. (*c*) AMI system operating on a UK farm powered by solar energy. UV, ultraviolet; SSD, solid-state drive; USB, universal serial bus; PCB, printed circuit board; LED, light-emitting diode.

An automated camera system for monitoring nocturnal insects requires a power source, a light source to attract insects to the trap location, a light source to illuminate the surface where insects land, a camera to record images, and a computer with storage to manage the system to schedule lighting and data capture, for example. One of the first complete systems was described by Bjerge *et al.* [19] and this design has been further modified as the Automated Monitoring of Insects (AMI) system (figure 2).

The system is designed for long-term (e.g. decadal) deployment, which is a vital feature for robust assessment of abundance trends that fluctuate markedly from year to year [22]. The use of solar power is important for widening the uptake of automated monitoring systems for insects, e.g. for long-term deployment in areas of the globe that do not have mains/gridded electricity or stable power supply. Photovoltaic (PV) power potential can readily be estimated for any season and point on the globe based on solar radiation, temperature, efficiency and orientation of PV systems, although environmental factors (e.g. clouds, snow, dirt, vegetation shading) and other losses (e.g. through cables and other components) will reduce the final power supply available for running systems.

Unlike traditional monitoring systems, where sampling is strongly limited by human resources [6], automated systems can operate perpetually given power, storage or bandwidth and basic maintenance. This is a major strength; continuous imaging makes it easier to derive the diel activity of different moth species, something previously achieved only through intensive manual sampling [23,24]. However, the potential for continuous imaging raises dilemmas related to trap scheduling. Specifically, choices around hours or days of operation, image capture frequency, and on-board processing routines (e.g. compression, object detection and classification) reflect a trade-off between power consumption and storage, disturbance of insects, and the quantity of ecological information captured. Furthermore, the use of light to attract moths can have severe sublethal impacts, affecting mate-location, egg-laying, predation risk or foraging behaviour [25] and could ultimately distort the demographic trends we seek to quantify using automated systems [26].

An effective approach to manage the inevitable power and data storage constraints of automated systems is to make use of an integrated, single-board computer and its significant on-board processing power to process and analyse data closer to the point where it is created and to telemeter results in real-time. The major motivations for such edge computing for insect monitoring are likely to be scientific, including adaptive sampling and actionable insights from data by making autonomous operational decisions (e.g. Edge AI [27]).

The continued evolution of automated camera systems will inevitably lead to a diversity of hardware systems and how they are deployed (e.g. locations and scheduling of image capture; quality of image capture, which will affect subsequent image detection and classification). Practical constraints on locations suitable for deployment systems (e.g. that are accessible or secure from theft and/or disturbance) are also likely to result in spatio-temporal biases in datasets for some applications (e.g. assessing regional trends). Statistical models can describe and account for some of this variation, but to enable more effective analysis it is important to capture metadata of the main auxiliary variables that will help explain variation in biodiversity metrics of interest [28].

## 3. Machine learning workflow

Automated camera systems for nocturnal insects generate large quantities of image data and this is likely to grow rapidly. Effective data processing through deep learning and computer vision is therefore essential to realize the potential for widespread, long-term monitoring [29].

**Figure 3.**
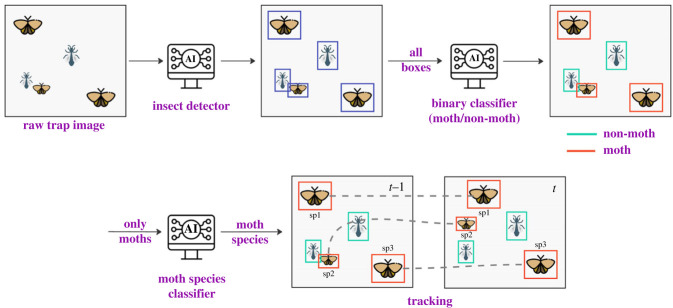
Machine learning workflow to analyse moth camera trap data. Step 1: insect detection; Step 2: moth/non-moth classification; Step 3: species classification; Step 4: tracking individual moths. sp, species. Reprinted with permission from [30].

Algorithms are in development for processing images from cameras operating at night, e.g. detection of individuals and classification [30]. These algorithms are motivated by requirements to support ecological applications, e.g. predictions with high accuracy across moth species, algorithms to work across hardware setups (e.g. using different camera and light sources) and geographical regions, while minimizing the requirement for manual labelling of training images. The analysis pipeline consists of a mixture of machine learning techniques and hard-coded approaches, encompassing four steps and a process for collating training images (figure 3). We discuss the challenges of such a pipeline below.

### (a) Training images

A major bottleneck to machine learning-based systems for automated insect monitoring is the need for labelled training data, which typically must be extensive. Some authors have created their own labelled datasets for particular sensor systems [31]. However, this is time-consuming and therefore has generally been limited to small sets of species, failing to capture the true complexity of insect biodiversity.

An alternative approach is domain adaptation (e.g. transfer from training to test use), offering the option to train on existing labelled images of insects taken by museums or citizen scientists, and then use these algorithms with images from the automated sensor system in question, with little to no additional labelling required. This makes it possible to leverage existing datasets, such as those indexed by the Global Biodiversity Information Facility (GBIF; https://www.gbif.org/), a global biodiversity portal used by museums, universities, and research institutes, as well as citizen science tools such as iNaturalist and e-Butterfly. GBIF indexes over 31 million images containing insects (as of October 2023).

A pipeline has been developed for obtaining data from GBIF for insect monitoring [30]. Given a list of moths, it is compared against the GBIF taxonomic backbone [32]. Duplicate images are removed (i.e the same image associated with more than one occurrence record), taxon names are synonymized into accepted names, and doubtful, fuzzy, and unmatched names are manually resolved to make a processed checklist for a given region of interest (e.g. a country, a bioclimatic region). The accepted taxon key for each species is then used to fetch images and metadata (such as location and publishing institution) using the Darwin Core Archive (DwC-A; GBIF) provided by GBIF [33]. Two example regions of Quebec–Vermont and UK–Denmark yielded over 600 000 and 990 000 images for 3157 and 3013 species, respectively.

### (b) Insect detection

The first stage of image-processing for camera trap data is detecting individual insects. This is a special case of the common computer vision problem of object detection, which consists of automatically inferring bounding boxes around objects of interest [34]. Relatively simple object detection algorithms not requiring machine learning have been used (e.g. [19]) but these approaches face challenges such as the high diversity of insects attracted to lights, the possibility of overlap between individual insects, inconsistent lighting, and the gradual contamination and discoloration of the surface where insects settle.

Jain *et al*. [30] therefore designed a lightweight deep learning approach for insect detection. To minimize the need for new labelled data, the approach starts with the Segment Anything Model (SAM [35]), which is already trained on generic image data. First, the SAM algorithm is used to segment insects from the camera images, from which proposed insect locations are derived. These insect detections are not perfect, but they are manually reviewed to correct errors, a faster process than starting from scratch. Next, these detected insects are randomly pasted on an empty background image with simple augmentations (flips and rotations) to create a large simulated labelled dataset. Finally, deep learning algorithms are trained on the synthetic dataset. This approach significantly outperforms previous models, in particular displaying a much improved ability to detect overlapping moths, while requiring very little manual labour in labelling. Enhanced labelled training datasets are required for further improvement of insect detection in real-world applications where unidentifiable (e.g. fast-moving insects that cause blurry images) or out-of-scope objects are encountered (e.g. vegetation and other objects entering the camera field of vision).

### (c) Moth/non-moth classification

Moths are not the only arthropods that are nocturnally active and attracted to lights, so an image classifier to differentiate between moths and other arthropods is useful for identifying sets of images for subsequent classification. Based on our personal experience of light trapping, groups with some positively phototactic species include a wide array of Coleoptera, Diptera, Hemiptera, Odonata, Orthoptera and Trichoptera, and the families Formicidae and Ichneumonidae within the Hymenoptera, as well as spiders (Araneae) and harvestmen (Opiliones). In Jain *et al*. [30], a binary classifier built to distinguish moths from non-moths using the ResNet-50 architecture [36] based on 350 000 images of these non-moth groups together with adult-stage images of moth species from multiple regions worldwide provided an accuracy of 96% on a held-out set from GBIF data. A future priority is to assess the accuracy on expert-annotated camera trap images, including an assessment of which moth species can be identified from images alone.

### (d) Species-level classification

The crux of a pipeline for processing images from automated camera systems is species-level identification, representing a fine-grained classification problem in computer vision, and a number of recent studies have concentrated on classification of moths [19,30,37]. With 160 000 named moth species in the world [38], and thousands potentially occurring even in a single location, this is a challenging task. Many closely related species may have only subtle, or in some cases, no visually distinguishing characteristics (e.g. where experts are unable to identify species from images alone). The number of training images per species also follows a long-tailed distribution, a severe case of ‘class imbalance’, i.e. some species have many images, while most have only a few (if any). Such imbalance can lead to inaccurate predictions for rare species or bias the model towards commonly occurring ones, even though the species with the least data may be of particular ecological interest.

In their introduction of automated camera systems for moths, Bjerge *et al*. [19] focused on classification of only a limited set of eight common species, constructing a balanced 2000-image labelled dataset of trap images to train their models. Using a ResNet architecture [36], the authors demonstrated an *F*1-score of 96.6%. Korsch *et al*. [37] designed a combination approach, with one classifier operating on the entire image and the other on specific parts selected based on salient features. The authors trained this algorithm using expert-labelled camera trap images, after pretraining on data from iNaturalist, and showed that even on a larger set of 200 species it achieved an accuracy of 93.13%.

Most recently, Jain *et al*. [30] aimed for identification of the full set of moth species present in regions of interest, aiming to develop approaches requiring no additional labelling of images for a new location or choice of hardware. To do so, their approach used GBIF training data instead of trap data, with separate models trained using regional checklists (e.g. countries or biogeographical regions). Since GBIF training data are different from the data used by automated systems, the performance of a naive method would degrade significantly. To minimize this effect, the authors applied a set of strong data augmentation operations: random crop, random horizontal flip, RandAugment [39], and a mixed-resolution augmentation that simulates the relatively low resolution of the images from the camera trap. To mitigate the impact of majority classes on the model, Jain *et al*. [30] also limited the number of training examples per species to 1000 randomly selected images. For the Quebec–Vermont region, with over 3000 species, these models still achieved a species-level accuracy of 86.1% on a held-out GBIF test set and an accuracy of 77.8% on a small expert-labelled set of camera trap data.

### (e) Tracking individual moths

To support estimates of the number of individuals of a given species, it is valuable to track individual moths between frames. The choice of tracking algorithms is a balance between speed and accuracy. Labelled tracking data from automated camera systems is a priority for formal evaluation of different algorithms, including classical techniques such as Kalman Filter used in other domains [40]. One approach to object tracking, building upon Bjerge *et al*. [19] is to assume that instances of the same individual moth in consecutive frames will likely be close to each other, similar in size, and similar in the feature space of the species classifier. The cost of the assignment between any two moth crops in two consecutive images can be calculated as a weighted combination of four factors: (1) intersection over union (IoU) [41], (2) ratio of crop sizes, (3) distance between box centres, and (4) similarity in classification model feature space. The lower the cost, the more likely the match. Linear sum assignment can optimally match moth crops, with unmatched individuals indicating that a moth either has appeared for the first time or has left the image.

## 4. Data infrastructure

Automated monitoring systems for insects will produce a large number of images to store and process that is orders of magnitude above what is typical for many ecological applications. The anticipated rapid increase in data volumes will lead to inevitable trade-offs in what (e.g. raw versus processed image data), where (e.g. centralized versus distributed), how (e.g. optimized for long- versus short-term storage) and who should store and manage data, with associated financial and environmental costs. Such challenges are common across big data domains and are best addressed through proactive data management and implementation of efficient, standardized and repeatable workflows through each stage of the application (figure 1). In response to this challenge, research data infrastructure is being developed to meet national and international requirements for species identification from digital media to facilitate large-scale biodiversity monitoring (e.g. [42]).

Data infrastructure requires efficient processing at all stages: data ingestion, transformation and curation. Although automated image-based monitoring systems for nocturnal insects are relatively homogeneous, there is diversity in the quality of raw images as sensor hardware continues to be developed. Data systems therefore need to ingest raw data from multiple sources in their original format, after which they are validated, cleaned and harmonized. This entails rectifying data anomalies, unifying disparate data formats, and enriching data with additional context or metadata. Lastly, data should be curated to deliver the data to end-users in a readily usable format. For efficient querying, performance and data management, unstructured data such as images or videos are best stored in a separate storage location.

A further layer of data infrastructure is end-user services to access computer vision algorithms for analysis of image data. This includes an algorithm repository where users can select which algorithm to use for species identification and access to high performance compute power. The AMI Data Companion is a software package that is working towards this aim, as a collaborative, open-source development [30] that runs machine learning models on large batches of images from insect camera traps, with the choice of models (e.g. for object detection, species classification) being configurable. It provides a graphical interface that can be run on all major desktop operating systems, as well as a command-line interface that can be run on multiple server nodes in parallel. Images and their metadata from field deployments of camera systems can be reviewed and visualized before any processing takes place—for example, to confirm that cameras ran as scheduled (through the night and on designated days) in order to capture details of sampling occasions. The software uses a relational database to organize raw image captures, their metadata and all results from the machine learning models. The database schema aligns with the Camtrap Data Package [43], with some additional tables and fields specific to the machine learning outputs and metadata. Results from any stage of the pipeline can be exported in a number of formats such as comma-separated values text files that can be readily imported into statistical analysis and visualization software.

## 5. Automated insect monitoring data analysis

Uncertainty and bias can arise at each step of the object detection and classification processes inherent to image analysis [44]. Beside these obvious sources of error, automated systems will also be afflicted by similar issues to when collecting occurrence or count data with conventional sampling methods. Although present on the study site during the sampling occasion, not all individuals or species will be detected by the system. Such imperfect detection of individuals is thus prone to lead to variations in the proportionality between counts of individuals and actual abundance across sampling schemes, survey methods or conditions, time or sites, as well as among species or phenotypes [45]. It is difficult to infer ecological relationships, including population trends, when sampling conditions vary, such as survey schedule, equipment (e.g. light intensity and wavelength spectrum, sheet dimensions), weather or darkness (e.g. moon-phase, light pollution) [46,47]. Standardizing and classifying equipment and procedures will partly alleviate these issues, but the parameters estimated from automated camera systems data such as site occupancy, abundance and species richness will remain biased owing to the imperfect detection of the species or individuals present during sampling [45]. Imperfect detection probability afflicts most datasets, but methods are available to control for it statistically.

One option is to model explicitly the ecological or state process (i.e. abundance or presence of one or multiple species) and the observation process (i.e. detection probability) [48]. Hierarchical models inspired from capture–mark–recapture theory have been developed to perform this task using data on unmarked individuals [49]. Such models are applicable to the analysis of automated camera systems. In essence, these models predict occurrence probability or abundance from histories of detections or counts replicated in at least two dimensions, such as time and space [50]. For example, detections can be recorded within different time intervals (using bouts of continuous footage or clusters of photographs) within each night to determine the flying period of one or more species at a site over one season. Deploying additional systems at different sites enables the comparison of site-specific species composition across the sampling period. How sampling occasions should be replicated and structured in time or space will ultimately depend on the research objectives [51].

The main advantage of modelling abundance and detectability simultaneously for multiple species is that we can use different covariates for each process. Variables that affect occurrence probability or counts can differ from those influencing detection probability. Like any regression model, however, covariates and interactions need to be chosen judiciously with respect to research goals to avoid spurious effects [52]. These models make assumptions that constrain the sampling design or research questions and possibly require additional parameters in case of departures from these assumptions. A key assumption of models simultaneously estimating the state and observation processes is that each datum must be recorded within a period during which the state of the population is considered closed. If the state variable of interest is occupancy, this means that there is no change in occupancy during the entire season (e.g. occupied sites remain occupied between first and last visits). By contrast, when the focus is on abundance, the closure assumption means that there is no change in abundance across visits. This assumption thus calls for operational definitions of site, visit (sampling occasion), and season (time elapsed between first and last visits), based on the research objectives and species ecology. Another important assumption of abundance models is that individuals cannot be counted more than once during a given sampling occasion. Violation of this assumption occurs if individual insects are not identified through natural or artificial marking when they leave a camera system and return to it during the same sampling occasion. To avoid this issue, we recommend using ‘snapshots’ rather than sessions of continuous recording; or to improve detectability, using temporal clusters of photographs as sampling occasions and determining the maximum number of individuals present in a single photograph of each cluster. Another assumption is that detections are independent among sampling occasions. This assumption will likely not hold because some light-attracted insects can ‘freeze’ on a sheet for long periods on a given night [53]. Adopting a lighting schedule whereby the sheet of the system is active and illuminated between periods of non-illumination on a given night may help alleviate this problem as well as the impacts of light pollution while improving detectability [54]. Minor modifications of the data-collecting protocol are often sufficient to meet the above-mentioned assumptions. Nonetheless, direct field observations on the presence and behaviour of individuals on and around the camera system could help refine sampling strategies and assess the validity of model assumptions and predictions. Valuable information could come from mark–recapture or removal of individuals attracted to the system or by alternative sampling methods deployed in the vicinity of the camera system [55].

An important implication of imperfect detection probability is that raw system data cannot be directly compared against raw data collected from conventional methods (e.g. lethal moth traps) for validation or calibration purposes (e.g. [55]). Informal comparisons may, however, help justify the suboptimal, yet sometimes essential pooling of data collected by different methods over time. That said, formal comparisons of the detection probability of automated systems and other methods can be performed with hierarchical models (e.g. by treating methods as different observers) when combining data that partly overlap in space and time. In fact, using a mixture of sampling methods in the same analysis may prove useful in situations where automated systems are limited in numbers and spatial extent, with risk-of bias for estimating regional trends [56]. A related approach seeing active development in the last decade consists in using integrated data models to combine different datasets, such as those from citizen science projects, to estimate demographic parameters [57].

Lastly, automated camera systems rely on algorithms for specimen detection, tracking and classification. A potential issue with machine learning classification is that algorithms will occasionally misidentify detected specimens, resulting in either false positives or false negatives [44,58]. Misidentifications may result from various non-mutually exclusive sources including incomplete species coverage in training set of images, low image quality and poor species discrimination capacity owing to algorithm quality or high visual similarity of species. Misidentification rate is expected to decrease with the improvement of imaging technology, classification algorithms, and training sets of images. Models controlling for imperfect detection are already available to incorporate misidentification probabilities [59]. Such models could, for instance, use a set of images validated by experts to inform misidentification probabilities from a portion of the processed images and ultimately estimate unbiased state variables of interest.

Recent developments in automated systems thus offer a number of possibilities that are compatible with many existing analytical approaches aimed at reducing bias inherent to wildlife surveys and monitoring programmes. With minor adaptations in sampling protocol, this compatibility will likely help automated systems revolutionize the estimation of distribution and population trends of insects.

## 6. Future perspectives

We present a comprehensive and integrated framework for automated image-based monitoring of nocturnal insects, from sensor development all the way to data analysis approaches specific to automated image-based monitoring of insects. Innovators and researchers have been narrowly focused in their endeavours to date; computer scientists have focused on algorithm developments, and ecologists and engineers have focused on hardware designs and field campaigns. Both communities are now realizing that their respective activities will fail to have impact unless supporting frameworks, workflows and tools are built around these activities. We therefore propose ten priorities towards a step-change in monitoring of nocturnal insects, a vital task in the face of rapid biodiversity loss from global threats such as climate change, pollution, invasive alien species and loss and degradation of habitats.

1. *Build a multidisciplinary team:* involve a dedicated group of experts from engineering, entomology, taxonomy and systematics, computer vision and computer sciences, ecology and statistics to collaborate on developing the framework.
2. *Camera network deployment*: co-create guidelines for the deployment of camera networks, considering factors such as site selection, camera maintenance, and power supply; align with existing regional and global standards for metadata.
3. *Data collection protocols*: as far as possible, standardize the methods for collecting image data, including camera specifications, positioning, and timing of image captures; collect detailed metadata to describe data collection from automated devices.
4. *Labelled image datasets*: build up and publish balanced image training and test datasets to improve and evaluate computer vision algorithms for insect detection and identification; engage entomologists to contribute to data collection and curation; work to integrate images with molecular sequence data.
5. *Image processing algorithms*: develop and validate computer vision algorithms for insect detection and identification; ensure these algorithms are adaptable to different species and geographical contexts, and provide predictions across taxon hierarchies; communicate limitations and uncertainty in model predictions.
6. *Data storage and management*: establish protocols for data storage, backup, archiving and access control; evaluate trade-offs between cost and value in data retention strategies; implement metadata standards for indexing images and associated information adhering to FAIR data principles [60].
7. *Statistical analysis and sampling design:* develop models that can integrate information on the probability of correctly classifying an observation into species and information from identifications validated by experts; evaluate performance of sensor systems to sample and quantify insect communities; determine optimal allocation of effort within and across sites to detect changes in ecological communities.
8. *Ethical considerations*: address ethical concerns related to data privacy, consent and the impact of monitoring on insect populations.
9. *Education and outreach*: develop training materials and workshops to exchange knowledge about the framework, its benefits, and best practices for monitoring.
10. *International collaboration*: encourage collaboration between countries and regions to create a global network for insect monitoring, sharing knowledge and best practices.

## Data Availability

This article has no additional data.
